# Supplementary material for: WordCommentsAnalyzer: A windows software tool for qualitative research
Source: F1000Res. 2018 Sep 4;7:536. Originally published 2018 May 3. [Version 2] doi: 10.12688/f1000research.14819.2 (PMC6137411; doi:10.12688/f1000research.14819.2)
Supplement: Supplementary file 3 [file f1000research-7-17614-s0002.tgz › b2a79496-4ada-47c4-9682-a99ce867836d.zip!/Eight Iranian Health Journals/Yafteh _28.docx]

© Yafteh  . This work is licensed under CC BY 4.0 (https://creativecommons.org/licenses/by/4.0/).

Volume & Issue: دوره 13 - شماره 4

Title: بررسی فراوانی تزریق خون و فرآورده‌های آن در مرکزآموزشی درمانی شهدای عشایر خرم‌آباد در سه ماهه دوم سال 1389

http://yafte.lums.ac.ir/article-1-633-fa.html

بحث و نتیجه‌گیری: در این مطالعه میزان 1/59 درصد از خون های درخواست داده شده مورد استفاده قرار نگرفت که با توجه هزینه های تولید، نگهداری، جداسازی فرآورده ها، انتقال تا محل بیمارستان و همچنین هزینه های کراسمچ اعم از آزمایشگاهی و نیروی انسانی، واقعی کردن درخواست ها بر مبنای نیازسنجی های علمی توصیه می شود. مواد و روش‌ها: مطالعه از نوع توصیفی تحلیلی است. تعداد نمونه‌ها 648 نفر برآورد شد و بیماران با درخواست خون و فرآورده‌های خونی در طی زمان مطالعه بررسی گردیدند. متغیرهای مورد بررسی در چک لیست ثبت و داده ها با استفاده از نرم افزار آماری SPSS آنالیز شد. یافته‌ها: نتایج نشان داد که در طول مطالعه 7746 بیمار بستری شد. از این تعداد برای 648 بیمار خون یا فرآورده خونی درخواست شد (8 درصد) که از این تعداد 265 نفر (9/40 درصد) تحت تزریق خون و فرآورده های آن قرار گرفتند. بیشترین فرآورده تزریق شده پک سل (3/71 درصد) بود. میانگین هموگلوبین بیماران قبل از تزریق g/dl49/9 بود . بیشترین علت تزریق خون کاهش مزمن هموگلوبین و بیشترین عمل جراحی منجر به تزریق خون ، جراحی شکستگی فمور بود. نسبت کراسمچ به تزریق خون معادل 1/1 محاسبه شد. مقدمه: خون یک منبع حیات بخش است که جانشین ندارد. محدودیت مدت زمان نگهداری و همچنین فرآیندهایی مانند آزمون های غربالگری سلامت خون، تعیین گروه و کراسمچ که مستلزم هزینه‌های پرسنلی و تجهیزات آزمایشگاهی می باشد، بر اهمیت نحوه صحیح درخواست و مصرف مناسب آن می‌افزاید. این مطالعه با هدف تعیین میزان تزریق خون و فرآورده های آن و اندازه گیری نسبت کراسمچ به تزریق خون انجام شد.

Title: ارتباط بین سطح سرمی پروتئین C و چاقی درکودکان

http://yafte.lums.ac.ir/article-1-637-fa.html

مقدمه: چاقی خود زمینه ساز بیماری‌های قلبی عروقی است. هدف از انجام این مطالعه بررسی وضعیت سرمی C‏RP به عنوان عامل التهابی پیشگویی کننده و سطح سرمی لیپیدهای خون دراطفال مبتلا به چاقی مرضی و اضافه وزن در مقایسه با اطفال نرمال می باشد. بحث و نتیجه‌گیری: بین ابتلا به چاقی ویا اضافه وزن در اطفال 8 تا 10 سال با CRP مثبت ارتباط معنی‌دار وجود دارد. یافته‌ها: در رابطه با عامل اصلی مورد بررسی در این پژوهش یعنی CRP،پس از تحلیل‌های به عمل آمده مشخص گردید که بین وضعیت سرمی CRPدر دو گروه اختلاف معنی‌دار وجود دارد به طوری که 31 % اطفال گروه بیمار که شامل کودکان مبتلا به چاقی و یا اضافه وزن بود دارای CRPمثبت بودند در حالی که این درصد در گروه کنترل صفر بود(001/0. (P<از بین چربی‌های خون، تنها بین متوسط مقادیر کلسترول در گروه CRPمثبت و CRP منفی اختلاف معنی‌دار وجود داشت به طوری که متوسط سطح سرمی کلسترول در گروه CRP مثبت ( mg/dl9/201 )از متوسط سطح سرمی کلسترول در گروه کنترل(mg/dl 1/183) بیشتر بود(1/2- = , T 04/.=P ). مواد و روش‌ها: در این مطالعه که به صورت مقطعی-تحلیلی انجام گردید، یک گروه 80 نفری از اطفال 8 تا 10 سال که چاق تشخیص داده شدند به عنوان گروه مورد در نظر گرفته شد و در مقابل گروه 80 نفری از اطفال در همان گروه سنی به عنوان گروه کنترل در نظر گرفته شد. سطح سرمی لیپیدهای خون اعم از LDL_C وHDL_C,TG,Cholestrol و وضعیت سرمی C‏RP به صورت کیفی در حالت ناشتا در هر دو گروه سنجش گردید. در نهایت اطلاعات به دست آمده بااستفاده از نرم افزار آماری SPSS و آزمون های مجذور کای T و فیشر تحلیل گردیدند.

Title: مقایسه اثر سفالکسین و فسفومایسین ترومتامول (منورول) در درمان عفونت‌های ادراری تحتانی زنان باردار

http://yafte.lums.ac.ir/article-1-638-fa.html

بحث و نتیجه‌گیری: با مشاهده عدم تفاوت بین سفالکسین و فسفومایسین، می‌توان از تک دوز فسفومایسین برای درمان باکتریوری بی‌علامت طی بارداری استفاده کرد. مواد و روش‌ها: در این مطالعه‌ مداخله ای 166 زن باردار ، که کشت ادراری مثبت داشتند (105< CFU) به طور تصادفی ، به دو گروه درمانی تقسیم شده وتحت درمان با سفالکسین و فسفومایسین ترومتامول قرار گرفتند. یک هفته ونیز یک ماه بعد از درمان ،کشت ادرار انجام گردید. یافته‌ها: شایع‌ترین میکروب‌ها در گروه سفالکسین: E.coli (8/87%)، استرپتوکوک (3/7%)، پروتئوس (4/2%) و کلبسیلا (4/2%) و در گروه فسفومایسین: E.coli (5/84%)، استرپتوکوک (5/9%)، پروتئوس (8/4%) و کلبسیلا (2/1%) بدون تفاوت معنی‌دار (737/0=p ) بودند. پس از یک هفته، موفقیت درمان در گروه سفالکسین 7/92%‌و در گروه فسفومایسین 7/91% محاسبه گردید (1=p ). مقادیر 15/15 odds ratio=و 01/1Relative risk= بدست آمدند. تمام کشت‌ها بعد از یک‌ماه منفی بودند. 2 نفر در گروه سفالکسین و 3 نفر در گروه فسفومایسین دچار اسهال شدند که تفاوت معنی‌دار نبود (68/0=p ). مقدمه: از آنجا که باکتریوری طی حاملگی با وزن کم هنگام تولد و نارسی نوزاد همراهی دارد، انتخاب درمان صحیح و مناسب اهمیت پیدا می‌کند. فسفومایسین ترومتامول جزو کم‌خطرترین داروها برای حاملگی است. مطالعه حاضر با هدف مقایسه این دارو با سفالکسین در نمونه‌ای از زنان حامله مراجعه کننده به بیمارستان شهدای تجریش طراحی گردید.

Title: بررسی میزان حساسیت و ویژگی سونوگرافی در تشخیص آپاندیسیت حاد دربیماران مشکوک مراجعه کننده به بیمارستان شهدای عشایر خرم‌آباد

http://yafte.lums.ac.ir/article-1-639-fa.html

مقدمه: التهاب حاد آپاندیس را آپاندیسیت گویند، که یکی از شایعترین اورژانس های جراحی است. آپاندیسیت باید به سرعت تشخیص داده شده و آپاندکتومی انجام گیرد تا از بوجود آمدن عوارضی همچون گانگرن و پارگی آپاندیس که منجربه پریتونیت، فلگمون وآبسه می شود ممانعت بعمل آید. چناچه بیماری مشکوک به آپاندیسیت حاد باشد جهت تشخیص قطعی باید اقدامات تشخیصی بیشتری صورت گیرد. با توجه به اینکه سونوگرافی به عنوان روش تشخیصی دقیقی برای ارزیابی پروسه های پاتولوژیک در هر قسمتی از بدن مورد قبول قرار گرفته است، لذا از میان اقدامات تشخیصی، سونوگرافی همراه با سایر علائم بالینی جهت تشخیص آپاندیس نقش بسزایی دارد. هدف از این مطالعه تعیین حساسیت و ویژگی سونوگرافی در تشخیص آپاندیسیت در بیماران مشکوک مراجعه کننده به بیمارستان شهدای عشایر خرم آباد بوده است. بحث و نتیجه‌گیری: با توجه به بررسی انجام شده، سونوگرافی قبل از جراحی آپاندیس می تواند در بیماران مشکوک به آپاندیسیت حاد، به تشخیص قطعی کمک نموده و از انجام عمل های بی مورد و نیز از تأخیر در درمان و بوقوع پیوستن عوارض ناشی از آن در بسیاری از موارد حاد جلوگیری کند یافته‌ها: از 131 مورد مشکوک به آپاندیسیت، 6 نفر سونوگرافی منفی داشتند و علائم آنها بهبود پیدا کرد و از مطالعه خارج شدند. در 125 بیمار آپاندکتومی شده دیگر، جواب سونوگرافی و پاتولوژی باهم مقایسه شدند، از 125 بیمار 88نفر (4/70%) سونوگرافی مثبت و 37 نفر (6/29%) سونوگرافی منفی داشتند. از مقایسه نتایج سونوگرافی و پاتولوژی، 72 بیمار مثبت حقیقی، 16 بیمار مثبت کاذب، 12 بیمار منفی حقیقی، 25 بیمار منفی کاذب بدست آمد.همچنین حساسیت74% و ویژگی آن 43% بدست آمد. مواد و روش‌ها: پژوهش انجام شده یک مطالعه توصیفی مقطعی است که در آن کلیه افراد شکم حادی که مشکوک به آپاندیسیت بوده و در مدت زمان 5 ماه به بیمارستان شهدای عشایر خرم آباد مراجعه کرده بودند مورد بررسی قرار گرفتند. اطلاعات مورد نیاز توسط پرسشنامه، معاینه افراد و جراحی توسط پزشک متخصص و مقایسه جواب سونوگرافی با پاتولوژی جمع آوری گردید و با استفاده از آزمون های آماری توصیفی و نرم افزار آماری SPSS مورد تجزیه و تحلیل قرار گرفت و سپس با مقایسه نتایج پاتولوژی و سونوگرافی موارد مثبت و منفی کاذب و حقیقی تعیین شد. حساسیت و ویژگی سونوگرافی نیز مشخص گردید.

Title: رابطه باورهای فراشناختی با اختلال افسردگی و اضطراب

http://yafte.lums.ac.ir/article-1-640-fa.html

مقدمه: باورهای فراشناختی با اختلال‌های هیجانی تعامل دارد واین موضوع می تواند در برنامه های آموزشی و مداخلات روان شناختی، مورداستفاده قرارگیرد. هدف این پژوهش، بررسی رابطه باورهای فراشناختی با اختلال افسردگی و اضطراب بود. مواد و روش‌ها: پژوهش حاضراز نوع همبستگی است. ابتدا تعداد 140 نفر بصورت خوشه ای انتخاب شدند. آنان سه پرسشنامه‌" باورهای فراشناختی کارت رایت-هاتون، اضطراب کتل و افسردگی بک" را تکمیل کردند. سپس داده های مربوط به 117 نفر( 84 مردو 33زن) با میانگین سنی 31 سال و 6 ماه وبا انحراف استاندارد 8/6 با استفاده از روش رگرسیون چند متغیری ، تحلیل شد. یافته‌ها: نتایج نشان داد که باورهای فراشناختی با اختلال افسردگی و اضطراب رابطه‌ای‌ مثبت دارد. میزان همبستگی باورهای فراشناختی با اضطراب 59/0و با افسردگی 67/0است. مؤلفه‌های باور در مورد غیرقابل کنترل بودن و باور در مورد کفایت شناختی می تواند،40 درصد از واریانس اضطراب و 50 درصداز واریانس افسردگی را پیش بینی کند. علاوه براین، مؤلفه‌های باور در مورد غیرقابل کنترل بودن و باور در مورد کفایت شناختی با"اضطراب صفت" و مؤلفه باور در مورد غیرقابل کنترل بودن با"اضطراب حالت" رابطه ای مثبت دارد. بحث و نتیجه‌گیری: نتایج این پژوهش همسو با تحقیقات پیشین، نشان می دهد که در نظر گرفتن باورهای فراشناختی در برنامه های آموزشی و مداخلات روان شناختی، می تواند در پیشگیری از اختلالات هیجانی ، کاهش و درمان آنها مؤثر باشد.

Title: مقایسه اثر سمیت برخی فسفرآمیدها بر روی رده سلول سرطان خون K562

http://yafte.lums.ac.ir/article-1-641-fa.html

مقدمه: فسفرآمیدهایی با چارچوب عمومی -C(O)NHP(O)- به علت کاربردهای مختلف در زمینه بیوشیمی و کشاورزی مورد توجه بسیاری قرارگرفته‌اند. این ترکیبات با ساختار شبه پپتیدی دارای خواص بیولوژیکی هستند و مهارکننده‌ آنزیم‌های استیل‌کولین‌استراز و اوره‌آز می‌باشند. از طرف دیگر ویژگی‌های دارویی و استریوشیمی حلقه‌های شش‌تایی شامل فسفر و همچنین نقش فعال آنها به عنوان آنالوگ‌های سیکلوفسفامید مورد توجه است. هدف از این مطالعه بررسی تاثیر موقعیت استخلاف نیترو روی حلقه فنیل همچنین قرار گرفتن گروه NH بین فنیل و کربونیل در ترکیبات اوره‌ای بر سمیت این ترکیبات در مقابل سلول سرطان خون K562 می باشد. مواد و روش‌ها: در این تحقیق فسفرآمیدهایی از دو دسته کرباسیل آمیدوفسفات (3-1) و N- فسفنیل‌اوره (6-4) با استخلاف نیترو با فرمول عمومیRC(O)NHP(O)NHCH2C(CH3)2CH2NH (R=2-NO2C6H4(1), 3-NO2C6H4(2), 4-NO2C6H4(3) (4-NO2C6H4(6), 3-NO2C6H4NH(5),2-NO2C6H4NH(4), بررسی و مقایسه گردید تا سمیت این ترکیبات در مقابل رده سلول سرطان خون K562 با استفاده از روش MTT ارزیابی شود. سپس ترکیبات با استفاده از نرم افزارGraph Pad PRISM version 5.0 مقایسه گردیدند.بحث و نتیجه‌گیری: از آنجا که پیوندهای هیدروژنی نقش مهمی در ویژگی‌های بیولوژیکی دارد، لذا پیشنهاد می‌شود که افزایش در پیوندهای هیدروژنی مشتقات با عامل اوره‌ای (6-4) ممکن است بیشتر بودن سمیت این مولکول‌ها ‌موثر باشد. علاوه بر این، اثرات فضایی و الکترونی استخلاف نیترو در موقعیت متا ممکن است در بیشتر بودن سمیت این مشتقات از دو موقعیت دیگر موثر باشد. یافته‌ها: نتایج بیولوژیکی به‌دست آمده در محیط آزمایشگاهی نشان دهنده سمیت بیشتر مشتقات با عامل اوره‌ای (6-4) نسبت به ترکیبات دیگر (3 - 1) در برابر سلول‌های سرطانی مطالعه شده است. همچنین نتایج بیانگر این است که مشتقات مورد مطالعه با موقعیت 3-NO2 (متا) (ترکیبات 2 و 5) سمیت احتمالی بیشتری از موقعیت‌های پارا ( ترکیبات 3 و 6) و اورتو(ترکیبات 1و 4) دارند.

Title: نقش هوش هیجانی در افزایش سلامت روانی مدیران مدارس

http://yafte.lums.ac.ir/article-1-642-fa.html

مقدمه: هدف اساسی تحقیق حاضر بررسی هوش هیجانی و رابطه آن با سلامت روانی، جنسیت، مقطع کاری مدیران بود. بحث و نتیجه‌گیری: با توجه به اینکه بین هوش هیجانی و سلامت روانی مدیران همبستگی مثبت وجود داشت؛ ضروری است مدیران ارشد آموزش و پرورش به منظور تقویت هوش هیجانی و مؤلفه های مربوط به آن دوره های کوتاه مدت آموزشی در زمینه علوم رفتاری را برنامه ریزی و تدارک نمایند تا از این طریق سلامت روانی مدیران به ویژه مدیران زن افزایش یابد. یافته‌ها: بر اساس یافته های تحقیق بین هوش هیجانی و سلامت روانی مدیران همبستگی مستقیم وجود داشت(01/0 >P). همچنین نتایج نشان داد که مدیران مرد در مقایسه با مدیران زن از نظر هوش هیجانی بالاتر بودند(01/0 >P)، اما بین مدیران مدارس ابتدایی، راهنمایی و متوسطه تفاوتی در ارتباط با متغیرهای هوش هیجانی مشاهده نشد. مواد و روش‌ها: نمونه های این تحقیق که توصیفی و از نوع همبستگی می‌باشد، شامل تعداد 130 نفر از مدیران مدارس شهر خرم آباد بود که با استفاده از روش نمونه گیری طبقه ای متناسب انتخاب شده بودند. برای گردآوری داده های مورد نظر از دو فرم پرسشنامه هوش هیجانی و سلامت روانی استفاده شد. داده های مورد نظر با استفاده از آزمون های ضریب همبستگی پیرسون، تی گروههای مستقل و تحلیل واریانس یک طرفه تجزیه و تحلیل شد.

Title: مقایسه عوامل خطر قلبی عروقی و شیوه زندگی سالم زنان خانه دار و شاغل در مناطق مرکزی ایران_ برنامه قلب سالم اصفهان

http://yafte.lums.ac.ir/article-1-643-fa.html

مقدمه: این مطالعه با هدف بررسی وضعیت عوامل خطر بیماری قلبی عروقی و شیوه زندگی سالم در زنان شاغل و خانه دار انجام شد. بحث و نتیجه‌گیری: با توجه به نتایج حاصله ، برنامه ای مداخلاتی جامع تری در زمینه شیوه زندگی سالم و کنترل عوامل خطر در این گروه باید انجام داد. یافته‌ها: از 6102 نفر، 5591 نفر(92%) خانه دار و بقیه شاغل بودند. شاغلین نسبت به افراد خانه دار وضعیت تغذیه نامطلوب و فعالیت فیزیکی مطلوب تری داشتند. استعمال دخانیات در دو گروه تفاوت نداشت. میانگین شاخص توده بدنی، لیپیدها و فشار خون ، تفاوت آماری معنی داری بین دو گروه یافت نشد. میزان فراوانی دیس لیپیدمی، چاقی و دیابت، در زنان خانه دار کمتر و خطر نسبی به طور مختصر در زنان شاغل بالاتر بود. مواد و روش‌ها: این مطالعه جزئی از برنامه قلب سالم اصفهان است . روش نمونه گیری خوشه ای چند مرحله ای در زنان بالای 20 سال بود. زنان به دو دسته شاغل و خانه دار تقسیم شدند. اطلاعات دموگرافیک، کیفیت خوراکی، فعالیت فیزیکی و دخانیات ثبت شد . فشار خون، شاخص های آنتروپومتریک، قند خون ناشتا، پروفایل چربی‌ها اندازه‌گیری شد. داده ها بااستفاده ازT-student, chi square, logistic regression پردازش شدند.

Title: بررسی تأثیر نگهداری سگ و گربه در ابتلا به توکسوکاریازیس

http://yafte.lums.ac.ir/article-1-644-fa.html

مقدمه: توکسوکاریازیس یک بیماری زئونوز بوده که بوسیله مهاجرت یا حضور لاروهای توکسوکارا کنیس یا توکسوکارا کتی در بافت‌ها یا اندام‌ها ایجاد می‌شود. انسان از طریق خوردن مواد غذایی آلوده به تخم انگل یا گوشت خام یا نیم پخته (میزبانان پاراتنیک) حاوی لارو انگل به توکسوکاریازیس مبتلا می‌شود. این مطالعه با هدف تعیین میزان آلودگی به توکسوکارا در صاحبان سگ و گربه و مقایسه آن با میزان آلودگی در افرادی که سابقه نگهداری از این حیوانات را نداشتند، انجام شد. بحث و نتیجه‌گیری: با توجه به نتایج این مطالعه، بخش عمده ای از صاحبان سگ و گربه در مواجهه با انگل بودند. آموزش صاحبان سگ و گربه و ارتقاء سطح آگاهی آنها در مورد راه‌های انتقال عفونت و بررسی آلودگی سگ و گربه و جلوگیری از آلودگی آنها توصیه می‌گردد. یافته‌ها: درمجموع تعداد 162 نمونه (94 نمونه درتماس و 68 نمونه بدون تماس باسگ وگربه) دارای آنتی بادی ضدتوکسوکارا بودند. ازمجموع 558 فرد نمونه گیری شده (235 نفر مذکر و323 نفر مونث) 396 نفر(96/70%) منفی و162 نفر (03/29%) مثبت بودند. ارتباط آماری معنی‌داری بین میزان آلودگی به توکسوکارا و جنسیت وجود نداشت (275/ 0P=). مواد و روش‌ها: در این مطالعه توصیفی– تحلیلی 558 نفر (405 نفر بدون تماس با سگ و گربه و153 نفر در تماس با سگ و گربه) از افراد بین 20-2 سال شهرستان تبریز در سال 1388 به روش تصادفی انتخاب و نمونه گیری گردیدند. نمونه های سرمی تهیه شده از آنان از نظر وجود آنتی بادی ضد توکسوکارا با استفاده از روش الایزا مورد بررسی قرارگرفت و داده ها با استفاده از آزمون آماری کای دو تجزیه و تحلیل گردید.

Title: اثر کفش طبی با راکر پاشنه تا پنجه بر میزان محدودیت دامنه حرکتی مفاصل پا و مچ

http://yafte.lums.ac.ir/article-1-645-fa.html

مقدمه: راکرها یکی از رایج ترین اصلاحات خارجی کفش‌های طبی هستند و در درمان مشکلات مفصل مچ و مفاصل میانی پا به کار می‌روند. هدف از انجام این مطالعه مشخص کردن میزان دقیق محدودیت حرکتی است که راکر پاشنه-تا-پنجه روی مفاصل پا و مچ ایجاد می‌کند. بحث و نتیجه‌گیری: این مطالعه نشان داد که این کفش طبی دامنه حرکتی مفاصل پا و مچ را در صفحه ساژیتال محدود می کند ولی تا حدودی باعث افزایش دامنه حرکتی این مفاصل در صفحه فرونتال می شود یافته‌ها: تفاوت میانگین دامنه حرکتی مفصل مچ با کفش راکر در مقایسه با کفش معمولی در صفحه ساژیتال کاهش( 005/0p= )و در صفحه فرونتال افزایش یافت( 028/0p= ). این تفاوت ها از نظر آماری معنی‌دار شد ولی در صفحه عرضی اختلاف میانگین( 203/0p= )معنی‌‌دار نشد. دامنه حرکتی مفصل میانی پا نیز در صفحه ساژیتال کاهش( 047/0p= )و در صفحه فرونتال( 013/0p= )افزایش یافت . این اختلاف از نظر آماری معنی‌دار شد. در صفحه عرضی نیز با کفش راکر کاهش دامنه حرکتی از نظر آماری معنی‌دار شد( 015/0p= ). مواد و روش‌ها: مطالعه حاضر از نوع نیمه تجربی و نمونه گیری غیر احتمالی در دسترس بود . نمونه آماری 15 دانشجوی دختر در گروه سنی 18 تا 24 سال ، با شاخص توده بدنی طبیعی و الگوی راه رفتن طبیعی بودند. ابزار مورد استفاده دستگاه تحلیل حرکت وایکان 470، با 6 دوربین مادون قرمز با فرکانس 60 هرتز بود. دامنه حرکتی مفاصل ضمن راه رفتن با استفاده از هر دو نوع کفش محاسبه شد و نتایج حاصل با استفاده از آزمون غیرپارامتری ویلکاکسون تحلیل شد.

Title: بررسی ارتباط دانسیته انرژی و امتیاز تنوع رژیم غذایی دانشجویان دختر دانشگاه علوم پزشکی اصفهان در سال تحصیلی 89-88

http://yafte.lums.ac.ir/article-1-646-fa.html

مقدمه: دانسیته انرژی و امتیاز تنوع رژیم غذایی از شاخص‌های مناسب برای بیان میزان کیفیت رژیم غذایی هستند. هدف از این مطالعه بررسی میزان این دو عامل کیفیت رژیم غذایی در دانشجویان اصفهانی است. بحث و نتیجه‌گیری: بر اساس نتایج، رابطه معکوسی بین دانسیته انرژی و امتیاز تنوع غذایی در بین دختران دانشجوی اصفهانی وجود دارد. هرچند که برای تأیید این یافته اجرای مطالعات آینده نگر ضروری است. یافته‌ها: میانگین دانسیته انرژی در این افراد 29/0±60/1 کیلوکالری بر گرم بود. افرادی که در بالاترین سهک دانسیته انرژی بودند، کمترین امتیاز تنوع رژیم غذایی را داشتند ( میانگین سهک اول: 92/1±61/9، میانگین سهک دوم: 22/1±98/6 و سهک سوم: 02/1±63/3 و05/0>p). همین روند درباره امتیاز تنوع سبزیجات (33/0±91/1، 26/0±54/1، 02/0±11/1 و 05/0>p) و میوه ها (72/0±98/1، 66/0±89/1، 3/0±44/1 و 05/0>p) نیز دیده شد. افرادی که در بالاترین سهک امتیاز تنوع رژیم غذایی قرار داشتند دارای کمترین میانگین دانسیته انرژی بودند (36/0±08/2، 31/0±69/1، 25/0±38/1 و 05/0> p). رابطه ای معکوس و معنی‌دار بین دانسیته انرژی رژیم غذایی و امتیاز تنوع غذایی دیده شد (03/0= p و3/0= r). مواد و روش‌ها: این مطالعه بر روی 289 دانشجوی دختر دانشگاه علوم پزشکی اصفهان که به روش تصادفی انتخاب شدند، انجام شد. برای ارزیابی دریافت معمول افراد از یک پرسش نامه تکرر مصرف مواد غذایی که قبلاً روایی آن سنجیده شده بود استفاده گردید. دانسیته انرژی رژیم غذایی با تقسیم انرژی دریافتی روزانه هر فرد (بر حسب کیلوکالری در روز) بر وزن کل غذای خورده شده توسط وی (به‌جز نوشیدنی‌ها) به دست آمد. برای محاسبه امتیاز تنوع رژیم غذایی به هر کدام از گروه‌های غذایی پنج گانه راهنمای هرم غذایی امتیاز جداگانه‌ای داده شد.

Title: نقش ترکیب KIR-HLA در پیوند سلول‌های بنیادی خون‌ساز

http://yafte.lums.ac.ir/article-1-647-fa.html

مقدمه: پیوند سلول‌های بنیادی خون‌ساز (HSCT) آلوژنیک درمان با ارزشی برای لوسمی‌های حاد مقاوم به درمان ، لوسمی‌های با خطر بالای عود ، سندرم‌های میلو‌دیس‌پلاستیک(MDS) و لوسمی میلوئیدی مزمن است. نتیجه HSCT به عوامل متعددی از جمله مرحله بیماری، میزان تشابه آنتی ژن لکوسیتی انسان (HLA) بین دهنده و گیرنده ، رژیم آماده‌سازی و وقوع بیماری پیوند علیه میزبان (GVHD) بستگی دارد. مطالعات اخیر نشان داده ‌است که یکی دیگر از عوامل بالقوه تاثیرگذار بر نتیجه پیوند حضور سلول‌های کشنده طبیعی (NK) آلوری‌اکتیو مشتق از دهنده است. آلوری‌اکتیویتی سلول NK به عنوان ناسازگاری بین لیگاندهای پذیرنده شبه ایمونوگلوبولینی سلول کشنده (KIR) دهنده و گیرنده (مدل لیگاند- لیگاند)، یا به عنوان فقدان لیگاندهای KIR گیرنده برای KIR مهاری دهنده (مدل پذیرنده- لیگاند)، یا به عنوان ناسازگاری ژن های KIR بین دهنده و گیرنده (مدل ژن - ژن) تعریف شده است. اثرات ضد لوسمی آلوری‌اکتیویتی سلول NK ، شامل میزان کمتر عود ، رد پیوند و GVHD است که در نهایت به بقا کلی (OS) بیشتر منجر می‌گردد. با این وجود، اثرات آلوری‌اکتیویتی سلول NK بر روی نتیجه HSCT در بیماری‌های خونی بدخیم یک عنوان قابل بحث است.

Title: سندرم گلدنهار

http://yafte.lums.ac.ir/article-1-648-fa.html

بحث و نتیجه‌گیری: سندرم گلدنهار ناهنجاری مادرزادی است که عمدتاً صورت را گرفتار می کند ولی درگیری سایر ارگان ها باید در نظر گرفته شود.مشکلات قلبی عمده ترین علت مرگ در این بیماران است. معرفی مورد: بیمار نوزاد یک روزه دختر که از مادر 30ساله به ظاهر سالم با زایمان واژینال متولد شده بود. ناهنجاری های مادرزادی در معاینه فیزیکی جلب توجه می کرد و شامل میکروتی و آترزی کانال گوش راست، هیپرتلوریسم، میکروگناتی و یک شکاف واسط بین گوش راست و لب بود .سوفل قلبی در معاینه قلبی وجود داشت. مقدمه: همی فاسیال میکروزومی یا سندرم گلدنهار ناهنجاری مادرزادی است که تظاهرات اصلی آن شامل اختلال رشد یک‌طرفه گوش (آترزی کامل)، فک و گردن است.این سندرم با ناهنجاری سایر ارگان ها همراه است و از نظر فنوتیپی تنوع بسیاری دارد.
